# Supplementary material for: Fus3, as a Critical Kinase in MAPK Cascade, Regulates Aflatoxin Biosynthesis by Controlling the Substrate Supply in Aspergillus flavus, Rather than the Cluster Genes Modulation
Source: Microbiol Spectr. 2022 Feb 2;10(1):e01269-21. doi: 10.1128/spectrum.01269-21 (PMC8809346; doi:10.1128/spectrum.01269-21)
Supplement: SUPPLEMENTAL FILE 3 — Supplemental material. Download SPECTRUM01269-21_Supp_3_seq13.pdf, PDF file, 0.6 MB [file spectrum01269-21_supp_3_seq13.pdf]

Title:

Fus3, as a critical kinase in MAPK cascade, regulates aflatoxin biosynthesis by controlling the substrate supply in *Aspergillus flavus*, rather than the cluster genes modulation

Author names and affiliations:

Longxue Ma#, Xu Li#, Fuguo Xing*, Junning Ma, Xiaoyun Ma, Yiran Jiang

Institute of Food Science and Technology, Chinese Academy of Agricultural Sciences / Key Laboratory of Agro-products Quality and Safety Control in Storage and Transport Process, Ministry of Agriculture, Beijing 100193, P. R. China

# These authors contributed equally to this work.

*Corresponding Author

Fuguo Xing: Institute of Food Science and Technology, Chinese Academy of Agricultural Sciences, 2 Yuanmingyuan West Road, Haidian District, Beijing 100193, P. R. China

Tel: +86-10-62811868

E-mail: xingfuguo@caas.cn

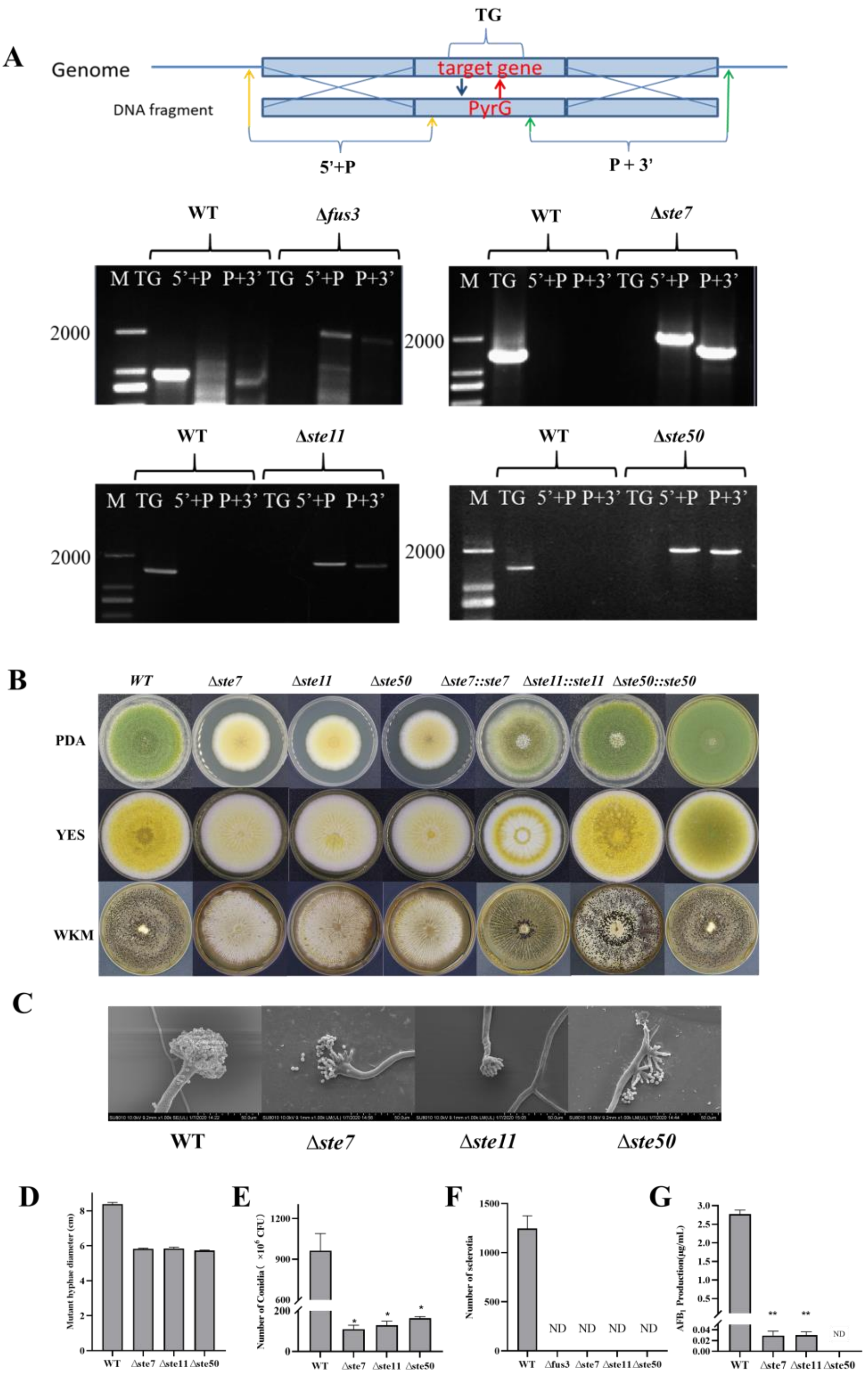

FIG S1 Construction and phenotypic variation of Δ*ste7*, Δ*ste11*, Δ*ste50* and their complements strains. (A) The null-deletion mode patten of mutants and the PCR examination of mutant strains; (B) WT, mutants and their complementary strains (Δ*ste7*::*ste7*, Δ*ste11*::*ste11*, and Δ*ste50*::*ste50*) were cultured on PDA, YES and WKM agar plates;

(C) the maldevelopment and sterility of conidia head and conidiophore of mutants under the scanning electron microscope; (D) the growth rate, (E) the conidia number, (F) the sclerotia number, and (G) the AFB_1 production of WT, mutants and their complementary strains. ND means "not detected", and * and ** show a significant difference at $p < 0.05$ and $p < 0.01$, respectively.

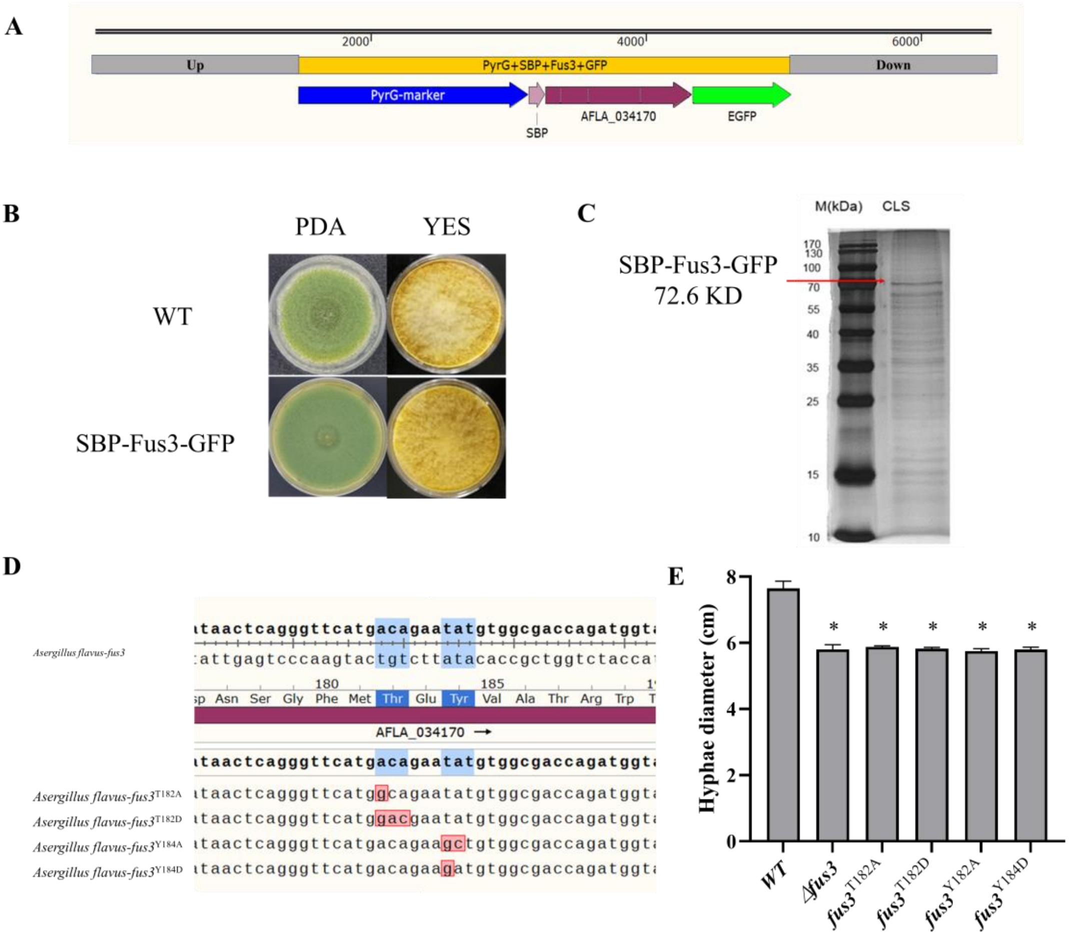

FIG S2 Verification and construction of both TAP-Fus3 and site-mutagenesis strains. (A) The genes' schematic design of a double-tagged protein construct for the Fus3 protein; (B) check the phenotype of WT and *sbp*::*fus3*::*gfp* strain on PDA and YES agar plates; (C) The SDS-PAGE electrophoresis result of SBP::Fus3::GFP proteins after tandem affinity purification (TAP); (D) Sequencing of the Fus3 point mutagenesis strains, including T182-A, T182D, Y184A, and Y184D; (E) the growth rate of WT, Δ*fus3*, *fus3*^{T182A}, *fus3*^{T182D}, *fus3*^{Y184A}, and *fus3*^{Y184D} on PDA agar plates.

Table S2 Comparisons of several TFs in *Δfus3* and WT by phosphoproteome and transcriptome analyses

Gene ID (AFLA_)	Gene	Gene description	Log2 (Δ*fus3*/WT)	significantly down-regulated phosphorylated peptides
004900		C6 transcription factor, putative	-0.26	3
006240	*Rum1*	PHD transcription factor (Rum1), putative	0.4	6
006980	*PalcA*	HLH transcription factor (PalcA), putative	0.81	2
014270		C6 transcription factor, putative	-1.35*	3
017640	*Rpn4*	C2H2 transcription factor (Rpn4), putative	-2.14*	1
017900	*FacB*	C6 transcription factor FacB/Cat8	0.27	2
018410		C2H2 transcription factor, putative	0.14	1
020210	*NsdD*	Sexual development transcription factor NsdD	1.38*	1
025030	*Hsf1*	Heat shock transcription factor Hsf1, putative	0.17	1
026100		Homeobox transcription factor, putative	-0.87	2
030500		PHD transcription factor, putative	0.55	1
030600		Forkhead transcription factor Fkh1/2, putative	-0.85	3
031340	*AtfA*	BZIP transcription factor (AtfA), putative	-1.19*	7
031450		CP2 transcription factor, putative	0.73	1
033160	*Sfp1*	C2H2 transcription factor (Sfp1), putative	-0.62	2
034540	*SrrA*	Stress response transcription factor SrrA/Skn7, putative	0.64	1
034700	*RfeD*	Transcription factor RfeD, putative	-0.45	6
040300		C6 transcription factor, putative	-2.72*	1
043760	*Con7*	C2H2 transcription factor (Con7), putative	-0.27	1
048650	*SteA*	Sexual development transcription factor SteA	1.12*	1
051100		C6 transcription factor, putative	-0.08	1
051340		C6 transcription factor, putative	0.04	1
066000		BZIP transcription factor, putative	0.76	1
069130		CBF/NF-Y family transcription factor, putative	0.18	1
070980		C6 transcription factor, putative	0.14	1
078390	*Sin3*	Transcription factor (Sin3), putative	0.41	1
078500		BZIP transcription factor, putative	1.13*	2

082520		C6 transcription factor, putative	0.12	2
084410		C6 transcription factor, putative	0.44	2
084970		C2H2 transcription factor, putative	-0.44	2
085880		BTB domain transcription factor, putative	-2.24*	2
086590	*Smp1*	Transcription factor smp1, putative	-0.55	3
088390	*Btf3*	Transcription factor btf3, putative	-0.79	2
090390		CBF/NF-Y family transcription factor, putative	-1.1*	1
091090		C6 transcription factor, putative	0.73	2
094460		C6 transcription factor, putative	0.66	1
105530		C6 transcription factor, putative	-1.98*	1
114960		Homeobox transcription factor, putative	0.97	1
122570		C6 transcription factor, putative	-0.11	2
123500		C6 transcription factor, putative	0.6	1
127920	*Crz1*	C2H2 transcription factor Crz1, putative	1.08*	3
128560	*PrnA*	C6 transcription factor (PrnA), putative	-0.78	2
131640	*Hpa3*	HLH transcription factor (Hpa3), putative	1.39*	2
132630		APSES transcription factor, putative	0.44	2
133380		Fungal specific transcription factor, putative	0.32	1
135110		HLH transcription factor, putative	0.36	1
136100	*AreB*	GATA transcription factor (AreB), putative	0.08	1
094010	*AtfB*	Basic leucine zipper (bZIP) transcription factor atfB	-1.58*	1
129340	*AP-1*	AP-1-like transcription factor yap1	0.69	2
049870	*AreA*	GATA transcriptional activator AreA	1.29*	1
029340	*Ngg1*	Transcriptional regulator Ngg1, putative	-0.02	2
054810	*RocA*	Transcriptional repressor TupA/RocA, putative	0.21	1

Transcriptome analyses were performed three biological replicates. Data was calculated with FPKM values. Significance was measured as the $p$adj < 0.05 and log2ratio ≥ 1 or ≤1.

Table S4 Fungal strains and plasmid used in this study

Strains/plasmid	Genotype	Reference
Aspergillus flavus TJES19.1	*Δku70; ΔpyrG*	From Yin Lab
Aspergillus flavus Δ*fus3*	*Δku70; Δfus3; pyrG*	This study
Aspergillus flavus Δ*ste7*	*Δku70; Δste7; pyrG*	This study
Aspergillus flavus Δ*ste11*	*Δku70; Δste11; pyrG*	This study
Aspergillus flavus Δ*ste50*	*Δku70; Δste50; pyrG*	This study
Aspergillus flavus Δ*fus3*::*fus3*	*Δku70; ΔpyrG; fus3*	This study
Aspergillus flavus Δ*ste7*::*ste7*	*Δku70; ΔpyrG; ste7*	This study
Aspergillus flavus Δ*ste11*::*ste11*	*Δku70; ΔpyrG; ste11*	This study
Aspergillus flavus Δ*ste50*::*ste50*	*Δku70; ΔpyrG; ste50*	This study
Aspergillus flavus $fus3^{T182A}$	*Δku70; Δfus3; pyrG::*$fus3^{T182A}$*ORF*	This study
Aspergillus flavus $fus3^{T182D}$	*Δku70; Δfus3; pyrG::*$fus3^{T182D}$*ORF*	This study
Aspergillus flavus $fus3^{Y184A}$	*Δku70; Δfus3; pyrG::*$fus3Y^{184A}$*ORF*	This study
Aspergillus flavus $fus3^{Y184D}$	*Δku70; Δfus3; pyrG::*$fus3Y^{184D}$*ORF*	This study
Aspergillus flavus sbp::*fus3*::*gfp*	*Δku70; Δfus3; pyrG::sbp::*$fus3^{ORF}$*::gfp*	This study
pMD18-T	Amp^R;	Our Lab
pYH-WA-pyrG-KI	Amp^R; *pyrG*	From Yin Lab
pMD18-T- *fus3*	Amp^R; *fus3ORF*	This study
pMD18-T-*fus3*-TAP	Amp^R; *SBP::fus3::GFP*	This study
pMD18-T- $fus3^{T182A}$	Amp^R; *fus3* ^{T182A}*ORF*	This study
pMD18-T- $fus3^{T182D}$	Amp^R; *fus3* ^{T182D}*ORF*	This study
pMD18-T- $fus3^{Y184A}$	Amp^R; *fus3* ^{Y184A}*ORF*	This study
pMD18-T- $fus3^{Y184D}$	Amp^R; *fus3* ^{Y184D}*ORF*	This study
Y2H Gold Yeast Strain	*MATa, trp1-901, leu2-3, 112, ura3-52, his3-200, gal4Δ, gal80Δ, LYS2::*$GAL1_{UAS}$-$Gal1_{TATA}$*-His3,* $GAL2_{UAS}$-$Gal2_{TATA}$*-Ade2* *URA3::*$MEL1_{UAS}$-$Mel1_{TATA}$ *AUR1-C MEL1*	From Chen and Guo Lab
pGADT7	Amp^R; *LEU2; Gal4 AD*	From Chen and Guo Lab
pGBDT7	Kan^R; *TRP1; Gal4 BD*	
pGADT7-*fus3*	Amp^R; *LEU2; Gal4 AD; fus3ORF*	This study
pGADT7-*ste7*	Amp^R; *LEU2; Gal4 AD; ste7ORF*	This study
pGADT7-*ste11*	Amp^R; *LEU2; Gal4 AD; ste11ORF*	This study

pGADT7-*ste50*	Amp^R; *LEU2; Gal4 AD; ste50ORF*	This study
pGBDT7-*fus3*	Kan^R; *TRP1; Gal4 BD; fus3ORF*	This study
pGBDT7-*ste7*	Kan^R; *TRP1; Gal4 BD; ste7ORF*	This study
pGBDT7-*ste11*	Kan^R; *TRP1; Gal4 BD; ste11ORF*	This study
pGBDT7-*ste50*	Kan^R; *TRP1; Gal4 BD; ste50ORF*	This study
pGADT7-*fus3*^{T182A}	Amp^R; *LEU2; Gal4 AD;* $fus3^{T182A}ORF$	This study
pGADT7-*fus3*^{T182D}	Amp^R; *LEU2; Gal4 AD;* $fus3^{T182A}ORF$	This study
pGADT7-*fus3*^{Y184A}	Amp^R; *LEU2; Gal4 AD;* $fus3^{Y184A}ORF$	This study
pGADT7-*fus3*^{Y184D}	Amp^R; *LEU2; Gal4 AD;* $fus3^{Y184D}ORF$	This study

Table S5 Primers used in this study

Primer name	Sequence（5'~3'）	Description
PyrG-F	gagagttattctgtgtctga	Mutant strain construction and verification
PyrG-R	attctgtctgagaggaggc	
fus3-5F	cattaggaggcaattggcgctg	
fus3-5R	cgtcagacacagaataactctcggactaacagagatagcgaacgg	
fus3-3F	cagtgcctcctctcagacagaatctcagccagcgtaagattgtg	
fus3-3R	ggagagacaggaggagaagaagtatc	
fus3-CS-F	gggtccagtgattaggtatcatc	
fus3-CS-R	caggaggagaagaagtatctgc	
ste7-5F	ccgtcatagtcatcagccaactc	
ste7-5R	cgtcagacacagaataactctcgccgcaatcactgaaggatg	
ste7-3F	cagtgcctcctctcagacagaatgagtacatcactatcgcacaacaga	
ste7-3R	gcaggttctgaactggcgatg	
ste7-CS-F	catagtcatcagccaactcctcttc	
ste7-CS-R	gtgctctctaatcactctcctcc	
ste11-5F	cgcacctcattatggagctag	
ste11-5R	cgtcagacacagaataactctcgtccggcaataactatagatcac	
ste11-3F	cagtgcctcctctcagacagaatgcagtgtctgatcgagattgatg	
ste11-3R	cgagtagagcaaacgtatcagtc	
ste11-CS-F	cattatggagctagcagttgtc	
ste11-CS-R	gagcaaacgtatcagtccacc	
ste50-5F	ctggagattgatccgcaagagg	
ste50-5R	cgtcagacacagaataactctccgaggataaggaccgacacgacac	
ste50-3F	cagtgcctcctctcagacagaatcctgaacaagattcgtgacattac	
ste50-3R	ctgcggtagaatcggatagcgag	
ste50-CS-F	ggtggtcgttgtcgtcgttcg	
ste50-CS-R	gcggtagaatcggatagcgagg	
Check-5R	gtcacatcagcagagacggtaac	
Check-3F	gcttggacagcaataccagact	
fus3-YZ-F	cgctaatacggtctagttcggac	
fus3-YZ-R	ctccattgtcggtgtaccaag	
ste7-YZ-F	cgtgtcgatgccaaggagaac	
ste7-YZ-R	gagctgcacgaacaggcatag	
ste11-YZ-F	gttctcggaggcacaggatg	
ste11-YZ-R	ctcaatcgacttgctgcgcgg	
ste50-YZ-F	gaatggacagtcgaagagtgcg	
ste50-YZ-R	cagacagattatagcactccgcc	
fus3ORF-F	atggtgcagcaacttcctccc	Gene Complementary Strain Construction
fus3ORF-R	tcaccgcatgatctcctcgtag	
ste7ORF-F	atggccgaccaattcaaagctcg	
ste7ORF-R	ttagcgttgccatggtgttccc	
ste11ORF-F	atgctggcaaaagctacgtattc	
ste11ORF-R	tcatgcaattggcgtggcgag	
Com -ste50ORF-F	atgtctctccacacttcctac	
Com -ste50ORF-R	ttatagcactccgccggg	
Com -fus3ORF-5R	gggaggaagttgctgcaccatggactaacagagatagcgaacgg	
Com -fus3ORF-3F	ctacgaggagatcatgcggtgactcagccagcgtaagattgtg	
Com -ste7ORF-5R	gagctttgaattggtcggccatgccgcaatcactgaaggatg	
Com -ste7ORF-3F	gggaacaccatggcaacgctaagagtacatcactatcgcacaacaga	

Com -ste11ORF-5R	gaatacgtagcttttgccagcatgtccggcaataactatagatcac	
Com -ste11ORF-3F	ctcgccacgccaattgcatgagcagtgtctgatcgagattgatg	
Com -ste50-5R	gtaggaagtgtggagagacatcgaggataaggaccgacacgacac	
Com -ste50-3F	cccggcggagtgctataacctgaacaagattcgtgacattac	
pMD18-T-fus3ORF-F	ctctcagacagaatcccgggatggtgcagcaacttcctccc	For Point mutation strain construction
pMD18-T-fus3ORF-R	gcctgcaggtcgacgatatctcaccgcatgatctcctcgtag	
pMD18-T-fus3^{T182A}-F	gttcatggcagaatatgtggcg	
pMD18-T-fus3^{T182A}-R	cacatattctgccatgaaccctg	
pMD18-T-fus3^{T182D}-F	gttcatggacgaatatgtggcg	
pMD18-T-fus3^{T182D}-R	cacatattcgtccatgaaccctg	
pMD18-T-fus3^{Y184A}-F	gttcatgacagaagctgtggcg	
pMD18-T-fus3^{Y184A}-R	cacagcttctgtcatgaaccctg	
pMD18-T-fus3^{Y184D}-F	gttcatgacagaagatgtggcg	
pMD18-T-fus3^{Y184D}-R	cacatcttctgtcatgaaccctg	
pGADT-T7-fus3-F	gccatggaggccagtgaattcatggtgcagcaacttcctccc	For Yeast two-hybrid vector construction
pGADT-T7-fus3-R	gatgcccacccgggtggaattctcaccgcatgatctcctcgtag	
pGADT-T7-ste7-F	gccatggaggccagtgaattcatggccgaccaattcaaagctcg	
pGADT-T7-ste7-R	gatgcccacccgggtggaattcttagcgttgccatggtgttccc	
pGADT-T7-ste11-F	gccatggaggccagtgaattcatgctggcaaaagctacgtattc	
pGADT-T7-ste11-R	gatgcccacccgggtggaattctcatgcaattggcgtggcgag	
pGADT-T7-ste50-F	gccatggaggccagtgaattcatgtctctccacacttcctac	
pGADT-T7-ste50-R	gatgcccacccgggtggaattcttatagcactccgccggg	
pGBDT-T7-fus3-F	atggccatggaggccgaattcgacagcagacacaacgccatac	
pGBDT-T7-fus3-R	gtcgacggatccccgggaattcccagctcaagattgacaagcg	
pGBDT-T7-ste7-F	atggccatggaggccgaattcatggccgaccaattcaaagctcg	
pGBDT-T7-ste7-R	gtcgacggatccccgggaattcttagcgttgccatggtgttccc	
pGBDT-T7-ste11-F	atggccatggaggccgaattcatgctggcaaaagctacgtattc	
Sbp-Fus3-F	aattcgagctcggtacccgggatggacgagaagaccaccgg	For pMD18-T-fus3-TAP
Sbp-Fus3-R	aagttgctgcaccatcccgggggggctcgcgctgaccctgg	
Gfp-Fus3-F	agatcatgcggtgagatatcatggtgagcaagggcgaggagc	
Gfp-Fus3-R	gcctgcaggtcgacgatatcttacttgtacagctcgtccatgc	
PyrG-F	gagagttattctgtgtctga	Construct strain SBP::Fus3::GFP
PyrG-R	attctgtctgagaggaggc	
P-SFG-S-F	gcctcctctcagacagaatggtaccatggacgagaagaccaccg	
P-SFG-G-R	ttacttgtacagctcgtccatgcc	
fus3-5F	cattaggaggcaattggcgctg	
fus3-5R	cgtcagacacagaataactctcggactaacagagatagcgaacgg	
SBP-3’-F	tggacgagctgtacaagtaactcagccagcgtaagattgtg	
fus3-3R	ggagagacaggaggagaagaagtatc	

fus3-CS-F	gggtccagtgattaggtatcatc	
fus3-CS-R	caggaggagaagaagtatctgc	
actin-F:	atcgttcttgattccggcg	For RT-qPCR
actin-R:	atgtggtgaagctatggcc	
aflR-F:	cctttctcactactcgggttt	
aflR-R:	gcaggtaatcaataatgtcgg	
aflS-F:	ctcgatgcggcagtgtatct	
aflS-R:	acacctccacatgagccttg	
aflA-F:	catgctgttaacccccgact	
aflA-R:	aattgggctaggaaaccggg	
aflC-F:	tgcatggcgatgtggtagtt	
aflC-R:	gtaaggccgcggaagaaag	
aflE-F:	gtgtggaggaagtgatgcga	
aflE-R:	cggggtaagtccgttagctc	
aflF-F:	ggttcgatgtttgctgaggg	
aflF-R:	gggtgaggacgaattggctt	
aflG-F:	gcaccaacaattcggctctg	
aflG-R:	tgtggaagggtggaagatgc	
aflH-F:	accaggttgaccacgtcttg	
aflH-R:	cacgaggtgtagtagacgcc	
aflJ-F:	gcgtgatcagtcgtcaatgc	
aflJ-R:	caggatgagcggttggttct	
aflK-F:	gctgggcattccagtacgat	
aflK-R:	cccatcaactgactgtggct	
aflN-F:	caaggcgaggtgtttcctct	
aflN-R:	ggcaagtgggtgatccttga	
aflQ-F:	gcaccaacaattcggctctg	
aflQ-R:	tgtggaagggtggaagatgc	
aflU-F:	gtgagtgcctcaggaatgct	
aflU-R:	tggagcacggatatgatggc	
aflX-F:	agtcctcaacatagccgctg	
aflX-R:	tagtcccccaggtttgacga	
aflT-F:	gcgtccgcctatctactgac	
aflT-R:	gaaaatacccagggcgacga	
abaA-F:	actggcaaaaggaggtcgag	
abaA-R:	attcgaacggtctgctggtt	
brlA-F:	tctagcggggatgacctcaa	
brlA-R:	ccgaaggaagccaaaagtgc	
veA-F	taccaacgaccattgccg	
veA-R	gagtacggagctgccaat	
laeA-F:	aaaggttgctcgctggtaca	
laeA-R:	gacttctgacgaaatgcgcc	
velB-F	tacaattcctcggcatcg	
velB-R	agtaccgaagctggaatgg	
atfA-F	ttcgctcacgtctcctgc	
atfA-R	taatcgcttgagccagtcg	
atfB-F	caagatggagcaacagacc	
atfB-R	gttgaaggcatactgtgcc	
ap-1-F	atgagcgctatcccgagc	
ap-1-R	ttgacttggtcaatgggcc	
msnA-F	cgaggctgtgactttgcc	

msnA-R	ttgacaaactcgtcctccg
mtfA-F	cttcgtccgtgactagtgg
mtfA-R	aaacgtggtggacagagc
srrA-F	cagaacaacgaagattccg
srrA-R	tgtagcatgatctgatggtc
creA-F	gtccaactctaccatggcc
creA-R	ctggtgctccaaacgatgg
areA-F	tctcacccagcgagtctcc
areA-R	cctgatggcggtggagag
pacC-F	ccagggttatgctcacgg
pacC-R	cgactcgtatgatgcgtgg
farB-F	tatcgcgcagaatgacgg
farB-R	ttccgacttgagtatccgg
